# Island hitchhikers: pathogen agents of Madeira and Azores ticks

**DOI:** 10.1007/s00436-024-08278-y

**Published:** 2024-07-05

**Authors:** Fernanda Rosa, Carla Silva, Ricardo Rodrigues, Mariana Esteves-Vieira, Inês Barbosa, Sara Rosa, Deodália Dias, Francisco Pina-Martins

**Affiliations:** 1 Instituto Superior de Agronomia, Universidade de Lisboa, Tapada da Ajuda, 1349-017 Lisbon, Portugal; 2 Centro de Estudos Do Ambiente E Do Mar (CESAM), LA, Faculdade de Ciências, Universidade de Lisboa, Campo Grande, 1749-016 Lisbon, Portugal; 3 Faculdade de Ciências, Universidade de Lisboa, Campo Grande, 1749-016 Lisbon, Portugal; 4 MSD Animal Health Portugal, Quinta da Fonte, Ed Vasco da Gama,19, Paço de Arcos, Portugal; 5 Computational Biology and Population Genomics Group, cE3c - Center for Ecology, Evolution and Environmental Changes & CHANGE - Global Change and Sustainability Institute, Faculdade de Ciências, Universidade de Lisboa, Campo Grande, 1749-016 Lisbon, Portugal; 6 Departamento de Engenharia Química E Biológica, Escola Superior de Tecnologia Do Barreiro, Instituto Politécnico de Setúbal, Rua Américo da Silva Marinho, 2839-001 Lavradio, Portugal

**Keywords:** *Ixodes ventalloi*, *Hepatozoon silvestris*, Pets, Madeira Island, Azores Islands

## Abstract

Ticks are blood-sucking arthropods that can transmit pathogens to their host. As insular ecosystems can enhance tick-host interactions, this study aimed to understand tick diversity, pathogen presence, and their respective associations in the Azores and Madeira archipelagos. Unfed or partially engorged ticks (*n* = 120) were collected from 58 cats and dogs in the Azores (*n* = 41 specimens) and Madeira (*n* = 79 specimens) from November 2018 to March 2019. Vector identification was based on morphology and molecular criteria. For pathogen sequencing, *18S* gene fragment for *Babesia*/*Hepatozoon* and *gltA* for *Rickettsia* were performed. Sequence data was explored using BLAST and BLAST and phylogenetic inference tools. In the Azores, *Ixodes hexagonus*, *I. ventalloi*, and *Rhipicephalus sanguineus* (*n* = 6; 14.6%, *n* = 6; 14.6%, and *n* = 29; 70.7% respectively) were found and in Madeira *I. ricinus* and *R. sanguineus* (*n* = 78, 98.7%; and *n* = 1, 1.3%; respectively) were identified. Tick COI markers confirmed species highlighting confirmation of *R. sanguineus* s.s. and genotype A of *I. ventalloi.* In the Azores Islands, the detected *Rickettsia massiliae* was linked to *R. sanguineus* (dogs and cats) and *I. hexagonus* (dogs), and in Madeira Island, *R. monacensis* (dogs) and *Hepatozoon silvestris* (cats) were found associated with *I. ricinus.* Further, *I. ventalloi* presence in the Azores expands west its known range, and *Hepatozoon silvestris* in Madeira may suggest that *I. ricinus* could have a role as a potential vector. Finally, as *R. massiliae* and *R. monacensis* presence underlines public health risks, surveillance by health authorities is crucial as pathogen-tick interactions may drive disease spread, therefore monitoring remains pivotal for disease prevention.

**Supplementary Information:**

The online version contains supplementary material available at 10.1007/s00436-024-08278-y.

## Introduction

Ticks are obligatory hematophagous arthropods that can potentially parasitize terrestrial vertebrates and act as vectors of pathogens such as bacteria, viruses, protozoa, and helminths (Ramos et al. 2013; Sonenshine and Roe 2014; Estrada-Peña et al. 2017; de la Fuente et al. 2017). In addition to the direct impact caused by pathologies, ticks can also have an indirect negative economic impact on countries’ agro-industry, due to the reduction in meat and milk production as well as body injuries induced by blood spoliation and secondary infections in livestock (Jongejan and Uilenberg 2004; Eskezia 2016).

Ticks are considered among the most important pathogen vectors worldwide, surpassed only by mosquitoes (de la Fuente 2008). About 10% of known tick species are considered to have medical and veterinary importance (de la Fuente et al. 2017). This fact, associated with diseases they can cause, makes ticks the most common pathogen agent vectors in Europe (Jahfari et al. 2016; de la Fuente et al. 2017).

Azores and Madeira Atlantic archipelagos are part of Macaronesia (which also includes the Canary Islands and Cape Verde), an important biogeographical region due to its rich biodiversity, included in the Natura 2000 network. Previous studies based only on tick morphology have identified six different species in Madeira Island (de Almeida 1997): *Ixodes ricinus* (Linnaeus, 1758), *Haemaphysalis punctata* Canestrini and Fanzago, 1878, *Rhipicephalus bursa* Canestrini and Fanzago, 1878, *R. sanguineus* (Latreille, 1806), *R. (Boophilus*) *annulatu*s (Say, 1821), and *Hyalomma lusitanicum* Koch, 1844. In the Azores Islands, ten species are known: *I. frontalis* (Panzer, 1798), *I. hexagonus* Leach, 1815, *Dermacentor marginatus* (Sulzer, 1776), *H. lusitanicum*, *H. marginatum* Koch, 1844, *H. punctata*, *R. sanguineus*, *R. bursa*, *R. turanicus* Pomerantzev, 1940, and *R. (B.*) *annulatus* (Dias 1992; Borges et al. 2010; Literak et al. 2015).

Data on tick-borne diseases or pathogens in these regions are scarce and dispersed and mainly related to clinical reports on human disease. Nonetheless, in Madeira Island *Borrelia burgdorferi* s.l (*B. afzelii, B. burgdorferi* s.s. and *B. garinii*) (Matuschka et al. 1998), *Anaplasma phagocytophilum* (Santos et al. 2004; Carvalho et al. 2008), *Rickettsia monacensis*, *R. helvetica*, and *B. lusitaniae* have been detected in *I. ricinus* (Carvalho et al. 2008; de Sousa et al. 2012). In the Azores, *R. massiliae* was isolated from *R. sanguineus* collected in one dog (Foley and Reeves 2014); *B. turdi* was detected in *I. frontalis* from birds (Literak et al. 2015); *Phlebovirus* was isolated from *I. hexagonus* and *R. sanguineus* (Pimentel et al. 2019); *Hepatozoon* sp. was observed in reptiles (Rund et al. 2019); and *Theileria equi* was detected in imported horses (Baptista et al. 2013).

In this exploratory study, our main objectives are to provide a precise update on the diversity of ixodids and their pathogens in pets (dogs and cats) from the Madeira and Azores Islands. Specifically, we aim to:

- Identify the different species of ixodids present in these Atlantic islands
- Investigate potential links between molecularly identified pathogens and their respective putative vectors. These findings will allow health authorities to better pinpoint their efforts to monitor, minimize, and control potential outbreaks caused by these vectors and pathogens

## Material and methods

### Tick collection and morphological identification

Ticks included in this study were collected from 36 dogs and 22 cats that attended veterinary clinics located in urban areas for routine visits or other health issues. A total of 120 ixodids (*n* = 41 in the Azores Islands and *n* = 79 in Madeira Island) were sampled during the years 2018 and 2019. Ticks were collected with tick removal tweezers during external exams in the veterinary clinic, following international recommendations and rules for handling animals, and carried out with the permission of local authorities. These specimens became part of a tick collection housed at the University of Lisbon, the “Faculdade de Ciências and Instituto Superior de Agronomia Tick Collection Database” (FCISA). Specimens were stored in tubes (1 tube/host) with 70% ethanol, and kept at room temperature, until laboratory processing not longer than two weeks.

Ixodids were examined and photographed using a Leitz Laborlux K light microscope and a Leica M165C stereomicroscope coupled to a Leica DFC420 digital microscope camera with the Leica Application Suite (LAS) live measurement software (Microsystems 2009). Identification was based on external criteria of their morphology according to several authors (Heylen et al. 2014; Coimbra-Dores et al. 2016; Estrada-Peña et al. 2017; Hornok et al. 2017, 2021; Nava et al. 2018).

Mean values of ticks found in hosts were compared between islands using Wilcoxon signed rank tests implemented in R v4.3.1. The respective script can be found in in this GitLab repository.

### Molecular analyses

#### Vectors

Fed ticks (unengorged or partially engorged) tick specimens were washed twice in 70% ethanol to remove external contaminants and left to dry at room temperature. Subsequently, they were crushed and placed overnight in a Proteinase K solution (20mg/mL – NZYTech) at 55 °C.

Genomic DNA was extracted using a commercial kit (E.Z.N.A. Tissue DNA Isolation Kit, Omega Bio-tek) following the manufacturer’s guidelines. After extraction, DNA samples were quantified using a NanoDrop 1000 spectrophotometer (Thermo Fisher). All aliquots were stored at 4 °C until processing (not more than 1 week).

To validate their morphological identification, eighteen specimens (adults of *R. sanguineus, I. ventalloi* Gil Collado, 1936, and *I. ricinus* and nymphs of *I. hexagonus*) were amplified by conventional polymerase chain reaction (PCR) for the mitochondrial Cytochrome C Oxidase subunit I (*COI*) fragment using the following primers: LCO1490, 5′-GGTCAACAAATCATAAAGATATTGG-3′; HCO2198, 5′-TAAACTTCAGGGTGACCAAAAATCA-3′ (Folmer et al. 1994). Each reaction was performed with a total volume of 25 μL consisting of 12.5μL of Dreamtaq Green PCR Master Mix (2 ×) (Thermo Scientific), 0.4 μM of each primer and 3 μL of extracted DNA. The PCR protocol consisted of an initial denaturation step at 95 °C for 5 min, 35 cycles of 95 °C for 20 s, 45 °C for 45 s, and 72 °C for 45 s followed by a final extension step at 72 °C for 7 min.

#### Pathogens

For pathogen screening, all 120 ticks were tested by PCR amplification. *Babesia*/*Hepatozoon* 18S gene was amplified using 18SR/5′CCAGCAGCCGCGGTAATTC3′/18S F/5′CTTTCGCAGTAGTTYGTCTTTAACAAATCT3′ primers (Tabar et al. 2008). *Rickettsia gltA* gene was amplified using gltAF/5′CCTATGGCTATTATGCTTGC3′/gltAR/5′ATTGCAAAAAGTACAGTCAACA3′ primers (Roux et al. 1997). PCR amplifications were conducted in a final volume of 20 μL, comprising 10 μL of PerfeCta SYBR Green Supermix, Rox (Quanta bio), 0.3 μM of each primer, and 2.8 μL of template DNA.

Regarding the PCR conditions, for *Rickettsia* screening, an initial denaturation step at 95 °C for 5 min was followed by 45 cycles of a three-step amplification: 95 °C for 15 s, 51.6 °C for 30 s, and 65 °C for 45 s. For *Babesia/Hepatozoon* species, an initial denaturation step at 95 °C for 5 min was followed by 35 cycles of a three-step amplification: 95 °C for 15 s, 64 °C for 30 s, and 65 °C for 45 s.

To validate amplification efficiency, all PCR products with fragments with approximately 400 qb were verified by 2% agarose gel electrophoresis and purified with the commercial kit SureClean Plus (Bioline). Twenty-five successfully amplified samples (23 from Azores and 11 from Madeira) were outsourced for sequencing (STABVIDA, Portugal). Newly generated sequences were submitted to NCBI, and accession numbers can be consulted in Online Resource Tables 1 and 2.

### Genetic data analyses

Sequence trace files were quality-controlled and exported as FASTA sequences using SeqTrace v0.9.1 (Stucky 2012). Obtained sequences were split into three separate datasets (Online Resource Tables 3, 4, and 5), one containing the *18S* sequences of *Hepatozoon* samples, the other containing *Rickettsia*’s *gltA* sequences, and the third containing COI sequences of vector samples. Each obtained sequence was then queried against NCBI’s “nt” database using BLASTN v2.14.1 + (Altschul 1997) with default parameters to identify agent sequences and to confirm the identification of vector sequences.

For maximum reproducibility, analysis downstream of this point was fully automated using Snakemake (Mölder et al. 2021), resorting to a Docker image for process containerization. The workflow, scripts, and container build script can be found in this GitLab repository.

Each original dataset was complemented with sequences from NCBI (The respective accession numbers and references can be consulted in Online Resource Tables 3–5, one table per dataset). To infer phylogenies, sequences were aligned using mafft v7.520 (Katoh and Toh 2008) with “–auto” parameters and trimmed to the length of the shortest original sequence at the 5′ and 3′ ends. The best model for each alignment was selected using ModelTest-NG v0.1.7 (Darriba et al. 2020). The model was then used in RAxML-NG v1.1.0 (Kozlov et al. 2019) to infer a maximum likelihood (ML) best tree supported by 1000 Felsenstein bootstrap replicates (using a seed value of 12358). MrBayes v3.2.6 (Ronquist et al. 2012) was used to infer Bayesian trees, using default priors and 1,000,000 MCMC iterations. Inferred phylogenetic trees were plotted with a Python script using the Toytree library v2.0.5 (Eaton 2020).

## Results

**Fig. 1:**
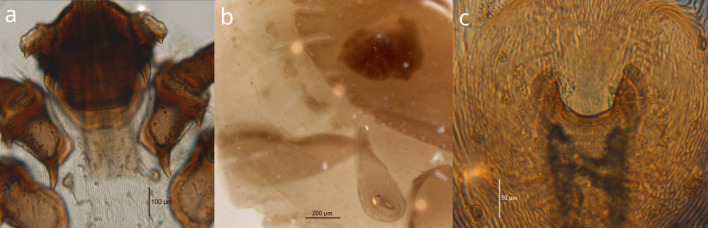
**a** *Ixodes ventalloi* ventral *basis capituli* with a horn shape oriented inwards and ventral face of the first article with a distinct spine; **b** *Rhipicephalus sanguineus* male ventral face with adanal plate, accessory adanal plate, and spiracular area with an ending inferior to half of the adjacent festoon width; **c** broad U-shape of female genitalia aperture with sclerites distant from each other

Morphological analyses of the sampled individuals revealed four different species of ticks: three in the Azores: *I. hexagonus*, *I. ventalloi*, and *R. sanguineus* (*n* = 6, 14.6%; *n* = 6, 14.6%; and *n* = 29, 70.7%; respectively), and two in Madeira: *I. ricinus*, and *R. sanguineus* (*n* = 78, 98.7%; and *n* = 1, 1.3%; respectively) (Fig. 1) (Table 1).

**Table 1:** Tick specimens collected in the studied islands

	Locality	*I. hexagonus*	*I. ricinus*		*I. ventalloi*		*R. sanguineus*		Total	***%***
		Nymph	Male	Female	Male	Female	Male	Female		
Azores Archipelagos	Faial Island	0	0	0	1	1	0	1	**3**	**2.5**
	São Miguel Island	6	0	0	1	3	0	4	**14**	**11.74**
	Terceira Island	0	0	0	0	0	10	14	**24**	**20.00**
	**Total**	**6**	**0**	0	**2**	4	**10**	**19**	**41**	
	**%**	**14.63**			**14.63**	**70.73**				
Madeira Island	Calheta	0	1	11	0	0	0	0	**12**	**10.0**
	Funchal	0	1	8	0	0	0	0	**9**	**7.5**
	Machico	0	0	8	0	0	0	0	**8**	**6.7**
	Ribeira Brava	0	1	5	0	0	0	0	**6**	**5.0**
	Santa Cruz	0	14	29	0	0	1	0	**44**	**36.7**
	**Total**	**0**	**17**	**61**	**0**	0	**1**	0	**79**	
	**%**		**98.73**				**1.27**			

The great majority of tick specimens were adults, except for the six specimens of *I. hexagonus*, which were nymphs (*n* = 6, 5.0%). Of the adult specimens, 70.8% (*n* = 85) were female and 24.2% (*n* = 29) male. Concerning tick hosts, 81 (67.5%) specimens were collected from dogs and 39 (32.5%) from cats. Despite the limited sample size, parasitical load showed higher values for Azorean hosts (M = 4.13, SD = 3.64 ticks per dog and M = 2.00, SD = 0.82 ticks per cat) than for Madeira (M = 1.68, SD = 1.49 ticks per dog and M = 1.72, SD = 0.59 tocks per cat). Wilcoxon signed-rank tests revealed differences in the mean number of ticks per dog between the Azores and Madeira Islands (*p* = 0.00979), but not in cats (*p* = 0.477). Specimens of *I. ricinus* and *R. sanguineus* were found in both hosts, *I. hexagonus* specimens were only found in dogs, whereas *I. ventalloi* specimens were collected exclusively in cats.

The performed BLAST search of the pathogens’ sequences against NCBI’s “nt” database revealed that all sequences matched with similar sequences from species they were morphologically identified as. In Madeira Island, *I. ricinus* sequences did so with 100% and 99.85% identity (MG432681.1), and the *R. sanguineus* sequences corresponded with 100% identity (MF426013.1). In the Azorean samples, the *I. hexagonus* sequences matched with 99.71% identity (MG432679.1), the *I. ventalloi* sequences had 99.23% identity (LC508372.1), and *R. sanguineus* showed 99.85% identity (MF426019.1).

**Fig. 2:**
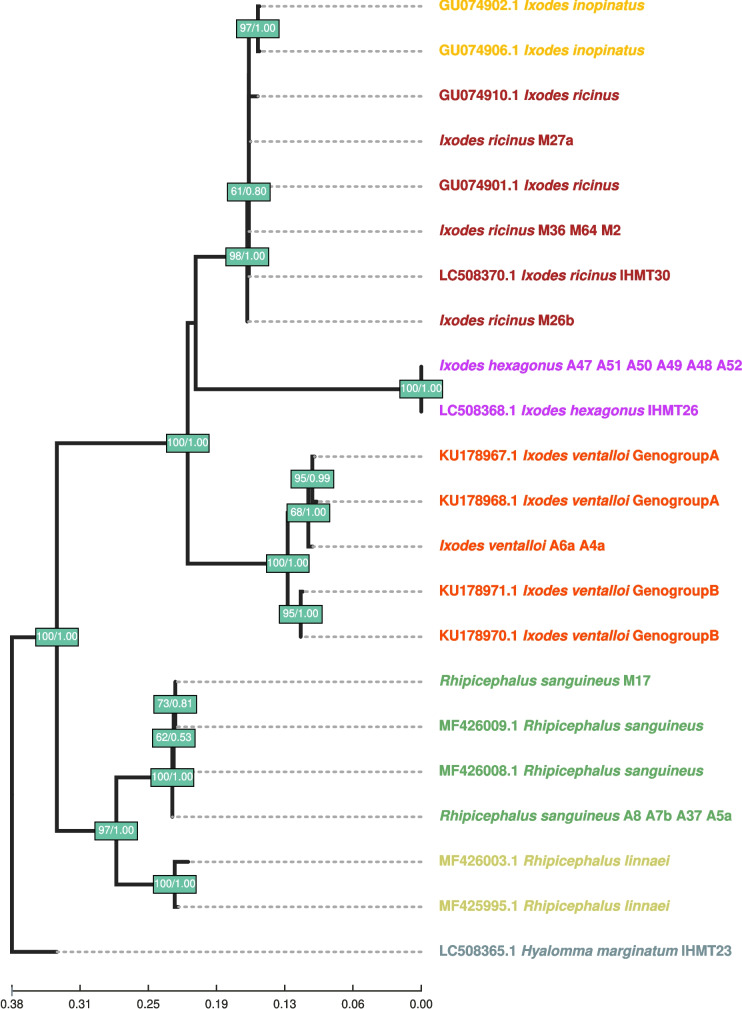
Maximum likelihood (ML)-based tick phylogram inferred from cytochrome oxidase I gene fragment. Samples’ labels of individuals sequenced for this work start with the species name, followed by the sample ID(s). Labels of sequences obtained from NCBI start with the respective accession number, followed by the species name

The obtained COI ML best tree shows that each species forms a well-supported monophyletic clade, except for *I. ricinus*, whose monophyletic clade has the lowest support of all groups (bootstrap = 61, posterior probability = 80), but is included in a monophyletic, well-supported clade with *I. inopinatus*. It is worth noting that Azorean *I. ventalloi* samples are closer to those of “Genogroup A” rather than “Genogroup B” samples. These monospecies clades contain both samples from this study and pre-identified samples from NCBI’s “nt” database, thus confirming morphological species identification results (Fig. 2).

In Madeira Island, *R. monacensis* was successfully amplified and sequenced from five *I. ricinus* samples (four females and one male). *Hepatozoon silvestris* sequences were obtained from two specimens of *I. ricinus* (one female and one male) (Fig. 4), both of which were also positive for *R. monacensis*.

In the Azorean archipelago, 23 ticks (*R. sanguineus*, *n* = 17; *I. hexagonus*, *n* = 6) were tested for pathogens. Eight ticks (34.78%) tested positive for *Rickettsia massilliae* in both *R. sanguineus* (*n* = 7/17, 41.18%) and *I. hexagonus* (*n* = 1/6, 16.67%). Most of these were found in Terceira Island (*n* = 6/17, 35.29%) *R. sanguineus*, but some detections occurred in S. Miguel (*n* = 1/17, 5.88% in *R. sanguineus*, and *n* = 1/6, 16.67% in *I. hexagonus*).

In Madeira Island, from the 11 tested ticks (*R. sanguineus*, *n* = 1; *I. ricinus*, *n* = 10), pathogen agents were only found in *I. ricinus*. *Hepatozoon sylvestris* (*n* = 2/11, 18.18%) was found only in Ribeira Brava (*n* = 2/10, 20.0%). *Rikettsia monocensis* (*n* = 6/10, 60.0%) was found in Ribeira Brava (*n* = 2/10, 20.00%), Sta Cruz (*n* = 2/10, 20.00%) Calheta (*n* = 1/10, 10.00%), and Machico (*n* = 1/10, 10.00%).

**Fig. 3:**
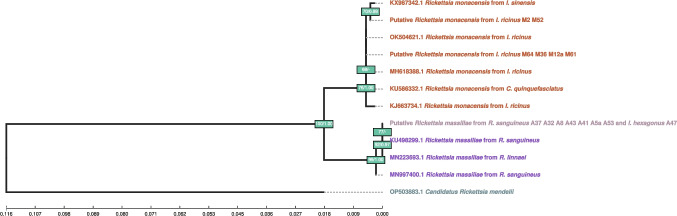
Maximum likelihood (ML) phylogram of *Rickettsia* (*gltA* gene fragment sequences). Labels of samples sequenced for this work start with the word “Putative,” followed by the assumed species name, and the vector’s species and sample ID. Sequences obtained from NCBI start with the respective accession number and otherwise follow the same scheme as the original samples’. Each agent species is represented in a different color. Nodes are labelled with Bootstrap support (BS) from ML, followed by posterior probability (PP) from Bayesian inference as BS/PP. A “-” indicates either BS < 40 or PP < 0.40. If both BS and PP are below this threshold, the node is unlabeled. *Candidatus Rickettsia mendelli* was used as an outgroup for tree rooting

In the Azores archipelago, only *R. massiliae* was successfully sequenced (one haplotype), but sequences were obtained from both *R. sanguineus* (three female and four male specimens) and *I. hexagonus* (six nymph) vectors (Fig. 3).

A BLAST search querying sequences from this study revealed that *Rickettsia* haplotypes (*n* = 3) from Madeira Island have a 100% identity with ten different *Rickettsia* sequences: the more frequent haplotype with four *R. monacensis* and one *Rickettsia sp.* sequences found in the “nt” database (GenBank)(MH618388.1, KU586332.1, KU961539.1, LN794217.1, AF141906.1, and LC060719.1) and the less frequent haplotype with four *Rickettsia* sp. sequences (LC060716.1, OP125492.1, OP125489.1, and AF140706.1). Likewise, the single haplotype found in the Azores was matched with 100% identity to three *R. massiliae* sequences (KU498299.1, CP003319.1, and KT032119.1) and one *Rickettsia* sp. sequence (U59720.1) from the “nt” database. Phylogenetic analyses of *Rickettsia* sequences revealed that samples from both species found in our study form well-supported monophyletic clades with sequences of their respective species from other studies, confirming what the BLAST search had already shown (Fig. 3).

**Fig. 4:**
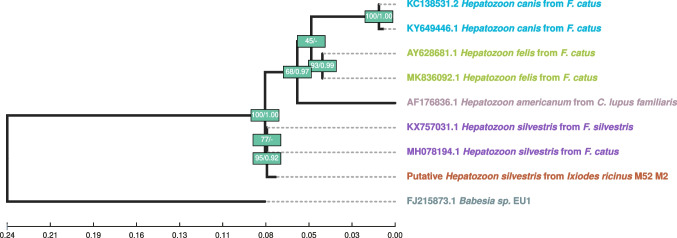
Maximum likelihood (ML) phylogram of *Hepatozoon* (*18S* gene fragment sequences). Labels of samples sequenced for this work start with the word “Putative,” followed by the assumed species name, and the vector’s species and sample ID. Sequences obtained from NCBI start with the respective accession number and otherwise follow the same scheme as the original samples’. Each agent species is represented in a different color. Nodes are labelled with bootstrap support (BS) from ML, followed by posterior probability (PP) from Bayesian inference as BS/PP. A “-” indicates whether BS < 40 or PP < 0.40. If both BS and PP are below this threshold, the node is unlabeled. *Babesia* sp. UN1 was used as an outgroup for tree rooting

An NCBI BLAST search revealed that obtained *Hepatozoon* sequences had a 99.47% identity with seven *H. silvestris* sequences (MH078194.1, MF614155.1, KY649445.1, KX757032.1, KX757031.1, OQ816783.1, and OQ207707.1), which led us to identify it as such. Phylogenetic analyses with other close *Hepatozoon* species (*H. canis* and *H. felis*) confirm this assessment (Fig. 4).

## Discussion

Although this work is of relatively small scale, and in no way a systematic study on the host, vector, and agent tick system in the Macaronesia, which is urgently needed, various important observations were made.

The presence of *I. ventalloi* (six specimens) in three of 11 analyzed cats, from Faial and S. Miguel Islands, confirms this vector’s presence in these islands. According to existing literature (Petney et al. 2017; Estrada-Peña et al. 2018; Santos and Santos-Silva 2019), this is the westernmost *I. ventalloi* that has ever been reported.

Phylogenetic analyses of *I. ventalloi* COI sequences confirmed its inclusion in “Genogroup A” (Latrofa et al. 2017), similar to specimens from mainland Portugal (Santos et al. 2018).

The Apicomplexa *H. silvestris*, previously found in wild and domestic cats in Central Europe (Hodžić et al. 2017; Hodžić and Alić 2023), was found in male and female *Ixodes ricinus* specimens in Madeira Island. This corroborates the results of (Duplan et al. 2018) which reported *H. silvestris* in fed *I. ricinus* in Wales (UK). These findings are insufficient to establish *I. ricinus* as biological vector (Hodžić and Alić 2023), but it adds more weight to that hypothesis, especially considering that males of this species exhibit blood-feeding behavior (Gray 1987). The fact that *Hepatozoon* spp. is known to cause severe, or even lethal pathologies in cats (Kegler et al. 2018; Simonato et al. 2022), coupled with the suspicion of its transmission by *I. ricinus* (Uiterwijk et al. 2023) highlight the importance of implementing systematic monitoring of this vector. Despite these molecular detections, none of the sampled hosts was reported to display any tick-borne disease symptoms by the veterinary clinics.

We also found evidence of two different *R. monacensis* haplotypes associated with *I. ricinus*, a known vector for various agents, from dogs in Madeira Island. It is worth noting that the endemic lizard species, *Teira dugesii*, is a known host for this tick species and its pathogens (de Sousa et al. 2012). As such, it is likely to play an important role in the dynamics of vector-borne diseases (Carvalho et al. 2008; de Sousa et al. 2012; Matuschka et al. 1998; Santos et al. 2004), not only as a vector, but also as a reservoir. This is a possible explanation for the relatively high prevalence (and diversity) of *R. monacensis* in Madeira hosts. A stable population of these agents that can infect pets has a high potential to become a threat to human health.

This study further identified the association of *R. massiliae* with *R. sanguineus* and *I. hexagonus* in the Azores Archipelago, albeit on different islands. *Rickettsia massiliae*’s association with *R. sanguineus* is known (Parola et al. 2013), but the agent’s presence in *I. hexagonus*, in new information, even though this species is a known competent vector of multiple pathogen agents (Jahfari et al. 2017). This unexpected association can either be attributed to the tick feeding on infected animals or to an adaptation of the SFG bacteria to a new vector. Here too, a larger, systematic study is required to fully understand this finding.

On another note, sequences from ticks identified as *R. sanguineus*, another tick species known as a competent pathogen vector (Solano-Gallego et al. 2008; Baneth et al. 2015; Snellgrove et al. 2020), form a monophyletic clade with *R. sanguineus* samples from NCBI (Coimbra-Dores et al. 2018). This places our samples in the same temperate lineage that can be found across Europe, North Africa, North, and South America (Burlini et al. 2010; Coimbra-Dores et al. 2018; Nava et al. 2018) and in the Canary Islands (Chitimia-Dobler et al. 2019). As it stands, this information rejects the hypothesis that the species found in the Azores and Madeira could be *R. linnaei* (Audouin, 1826) (Šlapeta et al. 2022), which can be found in tropical regions such as the Cape Verde Archipelago (Coimbra-Dores et al. 2018). Making this distinction is important, since these two species were only recently “upgraded” from lineages (Nava et al. 2018).

Even though our study suffers from the abovementioned limitations that prevent it from being considered representative of a global assessment in these insular ecosystems, it functions as a first insight into the characterization of insular vectors and agents. Addressing these limitations on a larger scale, a systematic approach may lead to finding further interesting associations with wider public health impact. Such monitoring is critical for developing effective disease prevention and mitigation measures and safeguarding human and animal health, especially considering the current fast-paced climate change scenario coupled with large-scale movements of people and animals.

## Conclusions

Here, we present the first observation of *I. ventalloi* (Genogroup A) in the Azores, which corresponds to its westernmost distribution range.

We also challenge assumptions by detecting two previously unknown associations: *R. massiliae* with *I. hexagonus* (Azores), and *H. silvestris* to *I. ricinus* (Madeira). Although the associations are confirmed, these ticks’ roles as vectors for the pathogens warrant further research.

Despite the limitations in sample size, sequencing, and short time period, this study highlights the importance of monitoring vectors and pathogens in a globalized, changing world. Our findings unveil more of the complex interactions between all these factors that require comprehensive strategies in evolving disease landscapes.

### Supplementary Information

Below is the link to the electronic supplementary material.

Supplementary file1 (XLSX 15 KB)

## Data Availability

All sequences are deposited in GeneBank, and the accession numbers are available in Online Resource Tables 1 and 2. The code used for data analyses can be found in this GitLab repository (https://gitlab.com/StuntsPT/ticks-and-agents). It is also mirrored in Zenodo, under 10.5281/zenodo.8319715.
